# Larrey, Father and Son

**Published:** 1859-08

**Authors:** 


### LARREY, FATHER AND SON.

The announcement of the appointment of Baron Larrey as surgeon-in-chief of the army of the Alps, now of Italy, has awakened an interest concerning the antecedents of this surgeon, which the paragraphs circulating in the journal do not satisfy.

The career of the elder, Jean Dominique Larrey, is so well known as to require little notice. He commenced his career as surgeon on board a French national vessel in 1787, and was sent to the coast of Newfoundland. Returning to France the following year he was appointed assistant surgeon in the army, and served his first campaign, under Marshal Luckner, on the Rhine, in 1792. From that time to the battle of Waterloo, in 1815, he was always engaged in military duties, mostly as surgeon-in-chief of the imperial guard. In Italy, Egypt, Syria, Germany, Russia, wherever the French arms were carried, was Larrey found. He died at Lyons, in 1842, at the age of 76, Member of the Institute, of the Royal Academy of Medicine, Commander of the Legion of Honor, Member of the Council of Health of the Army, and bearer of many other honorable titles besides, he rose to the highest social rank. His principal works are his Memoirs of Military Surgery, *Clinique Chirurgicale*, and *Relation Medicale des Campaignes et Voyages, from* 1815 *to* 1840.

No surgeon has left so great a reputation, both scientific and popular. It was he of whom Napoleon said, "he is the most virtuous man I have known," and to whom the people of France erected a bronze statue by penny subscription.

This beautiful work of art, from the hand of David, the sculptor, stands in the court yard of the Val-de-Grace Hospital at Paris.

The present surgeon-in-chief of the army of Italy, Hyppolite Larrey, is his only son. It is no disparagement of the father to say, that he is in all respects a worthy successor. I believe he has already won many of the posts of trust and honor held by the elder Larrey. He is surgeon-in-ordinary of the Emperor, Professor of Clinical Surgery at the Val-de-Grace Hospital, Member of the Imperial Academy of Medicine, Member of the Council of Health of the army, Surgeon-in-Chief of the army of Italy, etc. It is said that, before leaving Paris, he donated the house in which his father was born to the town in which it was situated, and appropriated a sufficient sum to yield $250 per year, for the purpose of keeping up a school for children in it. The association of the younger Larrey with the younger Napoleon has something of poetic justice not often met with in real life. May the career of both be as glorious, and more fortunate than that of their predecessors.

